# Heterogeneous components of lung adenocarcinomas confer distinct *EGFR* mutation and PD-L1 expression

**DOI:** 10.1186/s12885-020-6631-z

**Published:** 2020-02-24

**Authors:** Yiran Cai, Hongbo Wu, Xiaoqin Shi, Yujie Dong, Xiujun Chang, Li Zhang, Lijuan Zhou, Dan Su, Ming Yang

**Affiliations:** 1 Department of Pathology, Beijing Chest Hospital, Capital Medical University, 97 Beiguan Machang Rd. Tongzhou District, Beijing, 101147 China; 2 Department of Pathology, Shanghai General Hospital, Shanghai Jiaotong University School of Medicine, 100 Haining Rd, Hongkou District, Shanghai, 200080 China; 3 Department of Medical Oncology, Beijing Chest Hospital, Capital Medical University, 97 Beiguan Machang Rd. Tongzhou District, Beijing, 101147 China; 4 Department of Thoracic Surgery, Beijing Chest Hospital, Capital Medical University, 97 Beiguan Machang Rd. Tongzhou District, Beijing, 101147 China; 5 Shandong Provincial Key Laboratory of Radiation Oncology, Cancer Research Center, Shandong Cancer Hospital Affiliated to Shandong University, Shandong Academy of Medical Sciences, 440 Jiyan Rd. Huaiyin District, Jinan, China

**Keywords:** Lung adenocarcinoma, *EGFR* mutation, PD-L1, Heterogeneity

## Abstract

**Background:**

Lung adenocarcinoma (LAC) is composed of lepidic, papillary, mucinous, micropapillary and solid components in its parenchyma. Complex responses to therapeutics result from intratumoral heterogeneity. However, it remains confused that what components in a mixed LAC tumor are responsible to the heterogeneous *EGFR* mutation and PD-L1 expression.

**Methods:**

We investigated *EGFR* status via laser microdissection to capture spatially separated cancer cell subpopulations and digital droplet PCR to determine the abundance of *EGFR* sensitizing mutation and naïve T790M. Whilst, PD-L1 expression level via tumor proportion score (TPS) was evaluated by Ventana immunohistochemistry using SP263 antibody. PD-L1 expression levels were tiered in < 1, 1–49% and > =50% groups.

**Results:**

*EGFR* mutation harbored in 154 (59%) of 261 LAC patients and more frequently occurred in papillary, lepidic and micropapillary constituents. Higher levels of PD-L1 were found in LACs at stage III and IV (68.3%) versus those at stage I and II (31.7%) (*P* = 0.04). Solid predominant LACs (41.3%) expressed PD-L1 with TPS > =50%, versus mucinous and lepidic LACs (*P* < 0.01). LACs with solid constituents also had more positive proportion of PD-L1 protein. Cut-offs < 1, 1–49% or > =50% were associated with patients’ progression-free survival and longer in the < 1% group (22.9 month, 95% CI 17.6–28.2) (*P* < 0.05). LACs consisting of two constituents with PD-L1 TPS < 1% had a better prognosis than the groups with single component and more than two components (*P* < 0.05). Eighteen LACs (6.9%) had concomitantly deletion in exon 19 or L858R and naïve T790M mutation. The abundance of T790M varied diversely with sensitizing mutation. PD-L1 expression was not concordant in same components and usually negative in the *EGFR*-mutated constituents. Heterogeneous PD-L1 expression occurred in the vicinity of stromal tissues. 58.8, 29.4 and 11.8% in ALK positive LACs (*N* = 17) were found PD-L1 expression via cutoffs of < 1, 1–49% and > =50%, respectively (*P* > 0.05).

**Conclusion:**

Intratumoral genetic heterogeneity of LACs was demonstrated associated with histological patterns. Heterogeneous PD-L1 expression in higher level usually occurred in solid component both in *EGFR* mutated and *EGFR* wild-typed LACs. *EGFR* mutated LACs heterogeneously had sensitizing and resistant mutation and was accompanied with PD-L1 expression, but discordant among histological constituents. Immune checkpoint inhibitor combined with third generation *EGFR* tyrosine kinase inhibitor should be more effective to these LACs.

## Background

Lung cancer is a most common cause of cancer-related deaths in the world. Lung adenocarcinoma (LAC) is a prevalent histological type in non-small cell lung cancer (NSCLC) [1]. The treatment of lung cancer is individualized, and thus relied on the results of molecular biology assays and each patient’s histology [2]. Individual responses are now suspected to tumor heterogeneity and challenge personalized medicine and biomarker development [3]. The development of epidermal growth factor receptor tyrosine kinase inhibitors (*EGFR*-TKI) and immune check-point inhibitors have led a new era in lung cancer therapy. Meanwhile, The fact that *EGFR*-driven NSCLC inhibiting antitumor immunity through the activation of the PD-1/PD-L1 pathway has been demonstrated by preclinical studies. However, epidemiology studies suggest that *EGFR* mutant NSCLC is more likely to decrease PD-L1 expression. To palliate these controversies, intense studies focus on tumor heterogeneity, which tends to result in mixed responses (MR) to systemic *EGFR*-TKI and chemotherapy. However, the clinical significance and potential mechanisms remain to be testified. Physicians and oncologists turn on comprehensive therapies combined to targeted treatment and immune checkpoint inhibitors. Molecular discordances between primary and metastatic tumors differ among histological types [4]. A subset of patients with *EGFR*-mutant LACs fosters MRs to *EGFR*-TKIs. This uncertainty is suspected with intratumor heterogeneity (ITH). An MR may be an unfavorable prognostic factor and are suspected with tumor genetic heterogeneity [5]. Temporal/spatial heterogeneity between primary and metastatic tumors have only a limited forecast associated with markedly worsened outcomes [6]. The new classification has been reported as an independent predictor of overall survival [7]. LACs usually have mixed components (lepidic, acinar, papillary, solid and micropapillary) in tumor masses. Therefore, it is requisite to quantitatively evaluate histological components [1]. It have not been ascertained whether these outcomes reflect inappropriate use of targeted therapies or greater invasiveness of tumors with increased genomic instability results in generation of multiple subclones.

Laser capture microdissection (LCM) enables researchers to recognize tissue architecture and molecular characteristics. This method that helps investigate pathological changes on a molecular, cellular, or tissue level becomes more and more precise, whereas the sample can be available in smaller and smaller sizes. This study is designed to reveal the discordance of *EGFR* mutation in histological subtypes and the expression of PD-L1 in AC components and to investigate the potential effectiveness on targeted therapy and chemotherapy.

## Methods

### Patients

261 LAC patients between 2010 and 2017 were enrolled in this study and follow up to the end of 2017. Progression-free survival of each patient was evaluated in this study. LACs were histologically diagnosed based on the WHO classification (2015). Clinical stage were evaluated according to the 7th edition of the American Joint Committee for Cancer (AJCC) staging system [8], *EGFR* mutation test were carried on and sufficient specimens were used to assess PD-L1 expression level. Clinical data were obtained from the electronic medical record database from Beijing chest hospital and all patients provided written informed consent for the use of their tumor specimens.

### *EGFR* mutation and *ALK* fusion assay on heterogeneous components of LACs captured by LCM

The feature that cancer cells of the same genotype locate contiguously has been suggested on colorectal cancer via microsatellite instability [9]. Therefore, a sample will contain a genetically identical population of cancer cells if excised small enough from a tumor tissue. All 8 μm-thick FFPE sections from *EGFR* mutant patients who underwent surgical resection were stained with hematoxylin and eosin. The LMD 7000 microdissection system (Leica microsystems, Wetzlar, Germany) was used to capture pure cell subpopulations in target regions selected from *EGFR*-mutant samples according to the 2015 world health organization classification. Greater than 10^4^ cells in each area were captured, and 1 to 4 areas (according to the amount of tumor cells) were selectively obtained in each adenocarcinoma subtype from each section. Total DNA was extracted from each captured LCM sample via AmoyDx FFPE DNA/RNA kit (Spin Column, ADx-FF03; Amoy Diagnostics, Xiamen, China). Selected areas were tested for *EGFR* mutations by AmoyDx Adx-ARMS *EGFR* mutation kit (Cat. No Adx-EG01; Amoy Diagnostics, Xiamen, China). *ALK* fusion gene was detected by AmoyDx Adx-ARMS *ALK* fusion types (Cat. No ADX-AE02; Amoy Diagnostics, Xiamen, China).

### Digital PCR detection of *EGFR* mutations on LCM tissues

T790M, exon 19 deletions, and L858R mutations were assessed by QX-200TM ddPCR system (Bio-Rad, Hercules, CA, USA) according to the manufacturer’s instructions. A series of EGFR T790M mutation reference standards were prepared by using Human Genomic DNA, Female (Promega, US) and NCIH1975 Cell Line genomic DNA (Research DX, US) to determine cutoffs with the following mutation allele proportion of 0, 0.1, 1, 10 and 50%. Owing to NCIH1975 cell line genomic DNA is heterozygous for EGFR T790M mutation, it was used as 50% EGFR T790M mutation reference standard. Human Genomic DNA, Female (Promega, US) is regarded as negative EGFR T790M mutation reference standard. 0.1, 1 and 10% EGFR T790M mutation reference standard contained 0.2, 2 and 20% NCIH1975 Cell Line DNA, respectively. The final concentration of the above reference is 20 ng/lL.

Twenty μl ddPCR reaction system was loaded into an 8-channel droplet generation cartridge (Biorad, Milan, Italy); Emulsion was generated with 70 μL of QX200 Droplet generation oil (Biorad, Milan, Italy) and the cartridge loaded in the QX200TM Droplet Generator (Biorad, Milan, Italy). The emulsed droplets were then transferred to a 96-well plate and amplified by standard PCR using a Mastercycler® (Eppendorf). Cycling conditions consisted of a denaturizing step at 95 °C for 5 min, followed by 40 cycles of 95 °C for 30 s and 60 °C for 1 min.

### PD-L1 expression assessed by immunohistochemistry

All tumor sections were reviewed by Dr. Cai and Dr. Dong. Sections containing representative components were selected for PD-L1 immunohistochemical staining. PD-L1 (SP263) Rabbit Monoclonal Primary Antibody (Cat. No. 790–4905) and all other ancillary reagents, including VENTANA detection kits, and negative antibody (Cat. No. 790–4795) were procured from Roche Diagnostics GmbH (Mannheim, Germany). PD-L1 antibody produces membranous and/or cytoplasmic staining. PD-L1 protein was stained on the Ventana BenchMark XT with Ventana PD-L1 SP263 antibody. PD-L1 expression was evaluated on tumor cells (TC) by a three-tiered grading system on tumor proportion score (TPS): < 1, 1–49% and > =50%.

### Statistical analysis

All LAC components were quantitatively diagnosed in 5% increment of tumor cells on FFPE tissue sections and each component was evaluated. Non-parameter analyses were performed on skewly distributed data. Categorical variables were compared by crosstab using chi-square test. Survival analysis by Kaplan-Meier method was performed for different groups, with the use of the log-rank test. All statistical tests were two-sided, significant level was α = 0.05. Variables included in this model were age, sex, histology, clinical stage and *EGFR* status. All data were analyzed by using the Statistical Package for the Social Sciences software, version 25.0 (SPSS, Chicago, IL) and GraphPad Prism (version 7.01).

## Result

### Patient characteristics and Histopathological features

Demographic data of patients were summarized in Table 1. Patient ages ranged from 23 to 83 years (median 58 years). There were 96 patients at stage I (36.7%), 21 at stage II (8.1%), 79 at stage IIIA (30.3%) and 65 at stage IIIB and IV (24.9%).

**Table 1:** Clinico-pathological characteristics in 261 patients of lung adenocarcinomas

	N	%
Gender		
Male	131	50.2
Female	130	49.8
Histological types (predominant components)		
Acinar	90	34.5
Lepidic	14	5.4
Micropapillary	22	8.4
Mucinous	10	3.8
Papillary	66	25.3
Solid	59	22.6
*ALK* fusion gene		
Positive	17	6.5
Negative	244	93.5
*EGFR* Status		
Mutated	154	59
Wild-typed	107	41
Clinical Stage		
IA	69	26.4
IB	27	10.3
IIA	7	2.7
IIB	14	5.4
IIIA	79	30.3
IIIB+IV	65	24.9

All patients were diagnosed of invasive LACs. 114 (43.7%) had uniform pattern and 147 (56.3%) harbored at least two types of constituents. 154 LACs (59%) had *EGFR* mutation, including acinar (55.1%), lepidic (85.7%), micropapillary (63.6%), mucinous (9.1%), papillary (81.8%) and solid (40.7%). *EGFR* statuses in histological subtypes were significantly different and mutations were more prevalent in lepidic, micropapillary and papillary LACs (*P* < 0.01)。17 LACs (6.5%) were detected with *ALK* fusion gene. The median PFS time of 261 patients was 17.6 months (range 0.3–72 months).

### PD-L1 expression varied in LAC histological pattern and with tumor stage

**Fig. 1:**
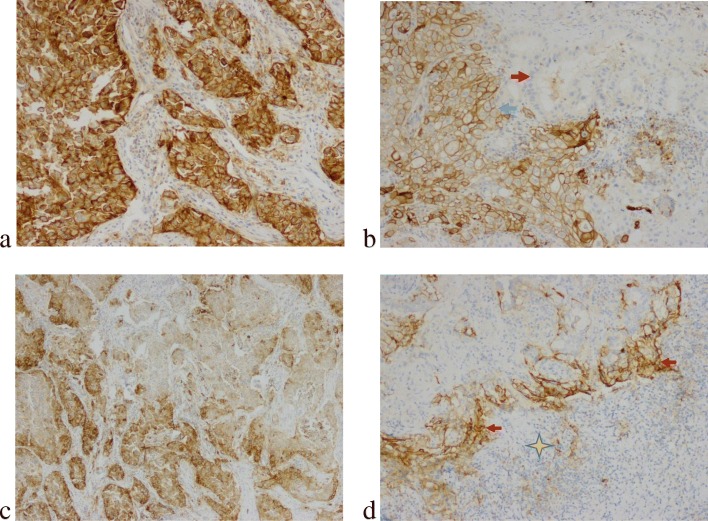
The typical cases for PD-L1 TPS. **a** PD-L1 was diffusely expressed in tumor cells. The TPS was almost 100% (× 200). **b** The TPS was 100% in solid area (blue arrow), but negative in acinar components (red arrow) (× 200). **c** The PD-L1 TPS was 70% with heterogeneous expression in the parenchyma with a single component; high TPS. Patchy positive staining was found in a solid area against stromal constituents (× 100). **d** Strong positive reaction to PD-L1 was stained in the peripheral cancer cells (red arrow) against to the stromal immune lymphocytes (asterisk) (× 200). TPS, tumor proportion score

**Fig. 2:**
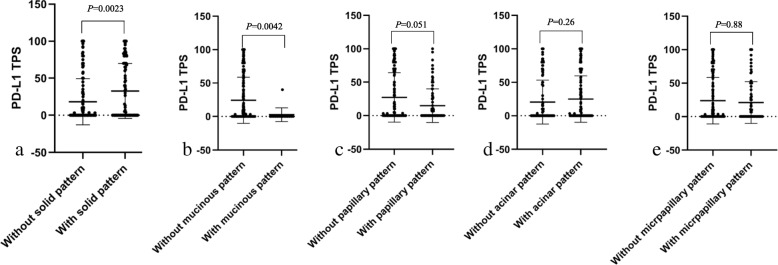
TPS level of tumor cells with different histological types. **a-b** The TPSs were compared among tumor cells with solid and mucinous patterns to those without corresponding patterns (*P* < 0.05). **c** The TPS in cancer cells without papillary structures was slightly higher than those with this structure (*P* = 0.051). **d-e** The TPSs were not associated with or without acinar and micropapillary components (*P* > 0.05). TPS, tumor proportion score

PD-L1 expression varied in LAC (Fig. 1). PD-L1 was noticed diffusely expressed in some types of tumor cells (Fig. 1a). However, patchy or heterogeneous expression was also detected in a same tumor mass (Fig. 1b), and even within the same component (Fig. 1c and d). Therefore, we compared PD-L1 expression level in different histological patterns (Fig. 2). PD-L1 TPS in LACs with solid component was higher than that in LACs without this pattern (*P* = 0.0023). LACs with mucinous components expressed in lower PD-L1 level compared to those without this pattern. Similarly, LACs harboring papillary pattern had slightly lower level of PD-L1 TPS than LACs without it (*P* = 0.051). Acinar and micropapillary constituents were not significantly associated with PD-L1 expression level.

We also assessed PD-L1 expression by using three tiered grading system: 1, 1–49% and > = 50% (Table 2). We assessed PD-L1 TPS in predominant components and found that histological types were associated with PD-L1 expression. In the group of PD-L1 TPS > =50%, Mucinous (0) and lepidic (1.6%) LACs were generally short of high-level of PD-L1 expression, whereas solid (41.3%) had the highest PD-L1 TPS (*P* < 0.01). Likewise, solid LAC was the type inclined to overexpress PD-L1 (41.3%, P < 0.01). Mixed LACs with the level of PD-L1 TPS > =50% were prevalent in LACs with two components than those with more than two components (*P* = 0.04). 144 patients were diagnosed at advanced stage (III and IV). We noticed that more than 50% cancer cells expressed PD-L1 in 43 LACs (68.3%) at advanced stage versus those (31.7%) at earlier stage (*P* = 0.04). PD-L1 TPS were not significantly associated with *EGFR* status (*P* = 0.7). In our study, 58.8, 29.4 and 11.8% in ALK positive lung adenocarcinomas (*N* = 17) were found PD-L1 expression tiered via cutoffs of < 1, 1–49% and > =50% tumor cells (*P* > 0.05).

**Table 2:** Association of expression of PD-L1 to clinicopathological characteristics in 261 patients of lung adenocarcinoma

		PD-L1 TPS (%)			*p* value
		< 1%	1–49%	> = 50%	
Sex	Male (*n* = 131)	75 (57.3)	24 (18.3)	32 (24.4)	0.92
	Female(*n* = 130)	72 (55.4)	27 (20.8)	31 (23.8)	
Clinical stage	I-II (*n* = 117)	74 (50.3)	23 (45.1)	20 (31.7)	0.04
	III + IV (*n* = 144)	73 (49.7)	28 (54.9)	43 (68.3)	
LAC predominant components	Acinar	57 (38.8)	15 (29.4)	18 (28.6)	< 0.01
	Lepidic	10 (6.8)	3 (5.9)	1 (1.6)	
	Microp	11 (7.5)	4 (7.8)	7 (11.1)	
	Mucinous	10 (6.8)	0 (0)	0 (0)	
	Papillary	39 (26.5)	16 (31.4)	11 (17.5)	
	Solid	20 (13.6)	13 (25.5)	26 (41.3)	
LACs	With uniform component (*n* = 114)	71 (48.3)	21 (41.2)	22 (34.9)	0.04*
	With two components (*n* = 104)	53 (36.1)	17 (33.3)	34 (54)	
	With > two components (*n* = 43)	23 (15.6)	13 (25.5)	7 (11.1)	
*EGFR* status	Mutated (*n* = 154)	84 (57.1)	30 (58.8)	40 (63.5)	0.7
	Wild-typed (*n* = 107)	63 (42.9)	21 (41.2)	23 (36.5)	

Abbreviations: *LAC* lung adenocarcinoma, *Microp* Miropapillary; *P, with some component compared with those without corresponding component

### PFS time of LAC patients with PD-L1 expression and histological subtypes

**Fig. 3:**
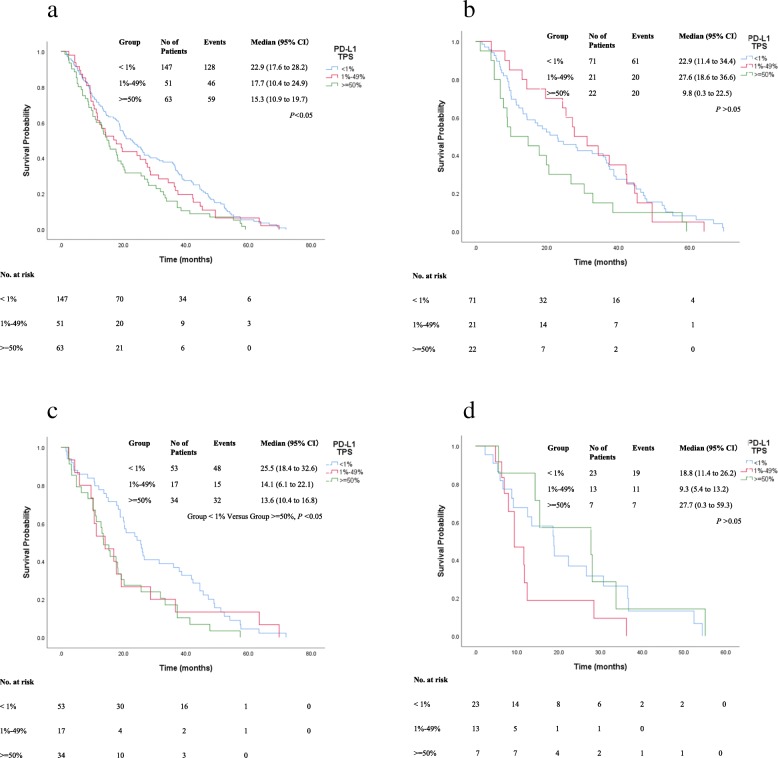
**a**-**d** Kaplan–Meier curve for progression-free survival (PFS) and PD-L1 expression TPS in LACs. **a** The median PFS survivals were compared in different PD-L1 TPS levels and the median PFS of group PD-L1 TPS < 1% was significantly longer than that of PD-L1 > =50% (P < 0.05). **b** The median PFS of groups PD-L1 TPS < 1, 1–49%, > = 50% in LACs with uniformed component was 22.9 months (95% CI 11.4–34.4), 27.6 months (95% CI 18.6–36.6) and 9.8 months (95% CI 0.3–22.5), respectively (P > 0.05). **c** The median PFS of groups PD-L1 TPS < 1, 1–49%, > = 50% in LACs with two components. PFS of group PD-L1 TPS < 1% was 25.5 months (95% CI 18.4–32.6), compared to that of group PD-L1 TPS > =50% with PFS of 13.6 months (95% CI 10.4–16.8 (P < 0.05). **d** The median PFS of groups PD-L1 TPS < 1, 1–49% and > =50% in LACs with more than two histological components. No significant difference was found in these three groups

**Fig. 4:**
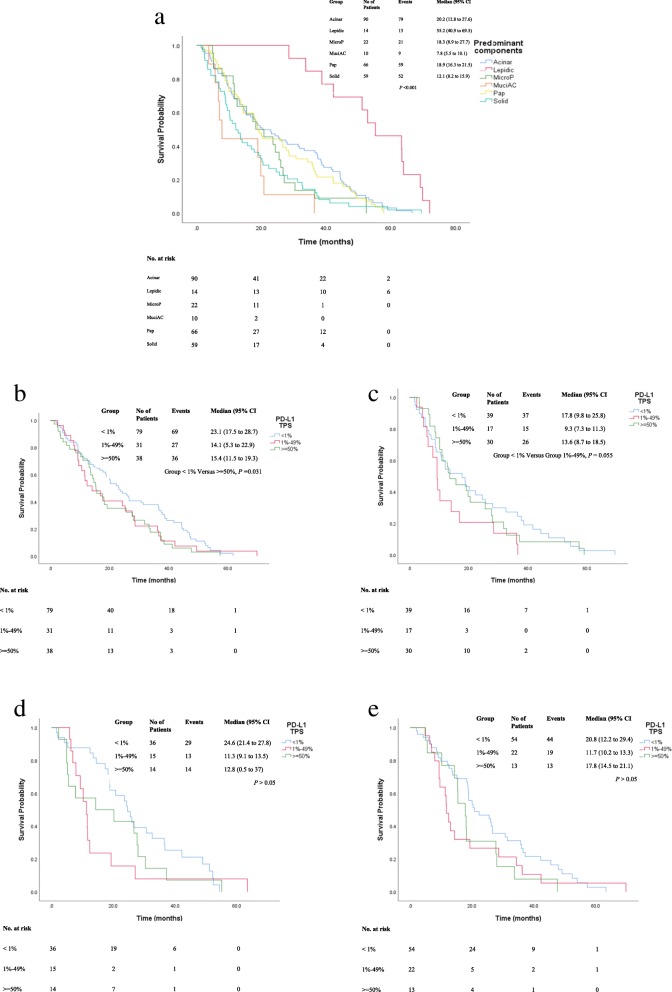
**a-e** Kaplan–Meier curve for progression-free survival (PFS) and PD-L1 expression TPS in LACs with different histological patterns. **a** The median PFS survivals were compared in different LAC subtypes. LACs with predominant lepidic components had longer PFS time than other subtypes (*P* < 0.001). **b** Among LACs with acinar pattern, the median PFS of group PD-L1 TPS < 1% was significantly longer than that of PD-L1 > =50% (*P* = 0.031). **c** Among LACs with solid pattern, the median PFS of group PD-L1 TPS < 1% was slightly longer than that of PD-L1 TPS 1–49% group (*P* = 0.055). **d** The median PFS of groups PD-L1 TPS < 1, 1–49%, > = 50% in LACs with micropapillary components. PFS of group PD-L1 TPS < 1% was 24.6 months (95% CI 21.4–27.8), although not significant longer than those with higher TPS (P > 0.05). **e** The median PFS of groups PD-L1 TPS < 1, 1–49% and > =50% in LACs with papillary components. Longer PFS time was found in the group of PD-L1 TPS < 1%, however, no significant difference was found in these groups (*P* > 0.05)

We firstly assessed PFS time of group < 1, 1–49% and > =50%. Of note, we found that high PD-L1 TPS (> = 50%) was associated with shorter PFS (median 15.3 versus 22.9 months, *p* < 0.05) in the entire cohort (Fig. 3a). To address whether the results were confounded by histological components, we observed the PFS time in different histological subtypes and found that PFS time was not significantly longer in three PD-L1 TPS groups among LACs with single histological pattern (*P* > 0.05) (Fig. 3b). PFS time was markedly longer in LACs with PD-L1 TPS < 1% (median 25.5 months) and harboring two histological components versus those with PD-L1 TPS > =50% (median 13.6 months, *P* < 0.05) (Fig. 3c). LACs with > = 2 histological components didn’t have significant difference in PFS time (*P* > 0.05) (Fig. 3d). Component-dependant heterogeneous PD-L1 expression were compared in LACs with such components as acinar, solid, micropapillary and papillary patterns. LACs with predominant lepidic components had longer PFS time (median 55.2 months) than other subtypes (*P* < 0.001) (Fig. 4a). Among LACs with acinar pattern, the median PFS of the group with PD-L1 TPS < 1% was significantly longer than that with PD-L1 TPS > =50% (*P* = 0.031) (Fig. 4b). The median PFS (17.8 months) of group PD-L1 TPS < 1% among LACs with solid pattern was slightly longer than that (9.3 months) of PD-L1 TPS 1–49% group (*P* = 0.055) (Fig. 4c). The median PFS time of groups PD-L1 TPS < 1% in LACs with micropapillary components was noticed, even though not significantly, longer than those with higher TPS (*P* > 0.05) (Fig. 4d). Similarly, the median PFS of groups PD-L1 TPS < 1%, was not significant difference with other groups (*P* > 0.05) (Fig. 4e).

### Intratumoral heterogeneous *EGFR* activating/naïve resistant mutations and different abundance in coaltered LACs

**Fig. 5:**
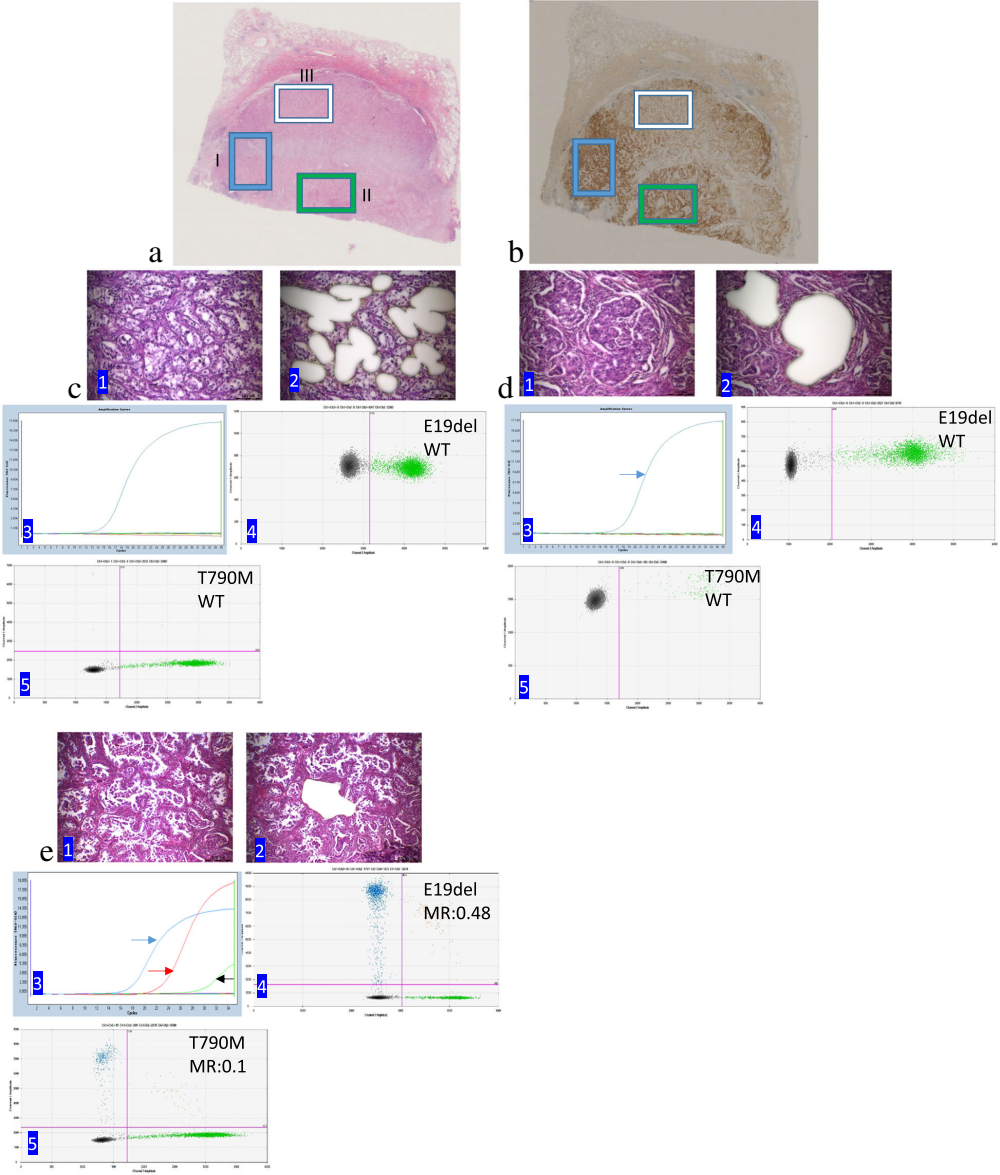
Pathologic and genetic characteristics and PD-L1 expression of one adenocarcinoma with *EGFR* coaltered exon 19 frame deletion (E19del) and T790M mutation by amplification refractory mutation system (ARMS) and digital droplet PCR (ddPCR)assays. **a** A paramount on the adenocarcinoma stained by hematoxylin and eosin. Area I-III were captured for genetic test. **b** A counterpart of section A were immunohistochemically stained for PD-L1 (SP263). Diffuse PD-L1 expression were found in area B-I and B-II, but negative in area B-III. (**c**1, **d**1 and **e**1) Acinar, solid and micropapillary components were microdisseced from area I, II, and III respectively. (**c**2, **d**2 and **e**2) The target tissues were captured by Laser microdissection. (**c**3-**e**3) *EGFR* mutation were screened by ARMS and wild-typed *EGFR* was found in acinar and solid area (blue arrow, postive control), whereas mutated in micropapillary area with E19del (red arrow) and T790M (black arrow). (**c**4-**e**4) E19del mutation was detected by ddPCR. Laser-captured tissues showed that E19del were absent in acinar and solid areas (**c**4 and **d**4, respectively), but present at micropapillary component with the abudance of 48% (**e**4). (**c**5-**e**5) T790M was also tested by ddPCR and acinar and solid components were negative for T790M (**c**5 and **d**5, respectively) whereas positive in micropapillary pattern with the abudance of 10% (**e**5). E19del, exon 19 frame deletion of *EGFR*; WT, wild type; MR, mutation ratio

We used amplification refraction mutation system and digital droplet PCR to analyze *EGFR* hotspot mutation. Eighteen patients had both *EGFR* sensitive (E19del/L858R) and naïve resistant mutation (T790M). We also observed their histological features, mutant *EGFR* abundance and expression level of PD-L1 to reveal the biological divergence of these components. All samples were further microdissected to determine the abundance of *EGFR* mutation in each histological component. We found that T790M mutation were generally accompanied with E19del/L858R except only one LAC harboring single T790M mutation (Table 3). Naïve resistant mutation predominantly coexisted with sensitizing mutations in micropapillary, papillary and lepidic components. Acinar, though, accreting with other components more frequently exhibited *EGFR* wild-typed in tumor parenchyma. As shown in Fig. 5, cancer cells captured in area I and II were of the acinar and solid subtype, which were negative for both sensitizing and resistant *EGFR* mutation, whereas cells in area III were positive for both sensitizing and resistant mutations. Of interest, the relative abundances of sensitizing and resistant mutations in the same dissected tumor focus varied irregularly. We observed that sensitizing/T790M mutation occurred predominantly in micropapillary and papillary components, whereas absent in acinar and solid components (Fig. 5a and b). In other words, tumor cells in same AC pattern from a same dissected region harbored heterogeneous cell population (Fig. 5c, d and e). These characteristics might be ignored by mass-based detection. The findings prompted that some tumor cells from this area were heterogeneous in their biological nature and might had inconsistent response to corresponding therapy.

**Table 3:** Expression of PD-L1 in different histological pattern with *EGFR* mutation

Isolated area	Histological components (%)	*EGFR* mutation (ARMS)	*EGFR* mutation in components (mutation, abundance%)	PD-L1 TPS (%)	PD-L1 differential expression
A	Papillary (60)	L858R, T790M	Papillary (L858R, 71; T790M, 9.7)	30	Papillary (Neg)
B	Acinar (40)		Acinar (Neg)		Acinar (80% pos)
A	Lepidic (80)	T790M	Lepidic (T790M) Microp (T790M)	10	Lepidic (Neg)
B	Microp (20)				Microp (50% pos)
A	Acinar (50)	E19del, T790M	Acinar (E19del, neg; T790M, neg)	70	Acinar (90% Pos)
B	Solid (35)		Solid (E19del, 48; T790M, 9.7)		Solid (70% Pos)
C	Microp (15)		Microp (E19del, neg;; T790M, neg)		Microp (Neg)
A	Acinar (60)	E19del, T790M	Acinar (E19del, neg; T790M, neg)	0	0
B	Microp (40)		Microp (E19del, 15.6; T790M, 9.5)		
A	Lepidic (100)	L858R, T790M	Lepidic (L858R, 5.7; T790M, 3.6)	0	0
A	Solid (70)	L858R, T790M	Solid (L858R, Neg; T790M, Neg)	80	Solid (90% pos)
B	Microp (30)		Microp (L858R, 66.1; T790M, 65.4)		
A	Acinar (65)	L858R, T790M	Acinar (L858R, neg; T790M, neg)	70	Acinar (Pos)
B	Papillary (35)		Papillary (L858R, 61.7; T790M, 25.8)		Papillary (10% pos)
A	Papillary	L858R, T790M	Papillary (L858R, 1.6; T790M, 1.2)	0	0
A	Papillary	L858R, T790M	Papillary (L858R, 50.9; T790M, 51.7)	0	0
A	Papillary	L858R, T790M	Papillary (L858R, 76.8; T790M, 85.3)	0	0
A	Papillary	E19del, T790M	Papillary (E19del, 44.3; T790M, 17.3)	0	0
A	Acinar	L858R, T790M	Acinar (L858R, 10.6; T790M, 9.94)	100	Acinar (Pos)
A	Acinar	L858R, T790M	Acinar (L858R, 5.7; T790M, 3.6)	0	0
A	Microp (90)	L858R, T790M	Microp (L858R, 10.5; T790M, 11.6)	0	0
B	Acinar (10)		Acinar (L858R, neg; T790M, neg)		
A	Acinar	E19del, T790M	Acinar (E19del, 8.5; T790M, 6.3)	0	0
A	Acinar (40)	L858R, T790M	Acinar (L858R, 17.3; T790M, 4.3)	30	Acinar (Pos)
B	Papillary (60)		Papillary (L858R, 56.3; T790M, 13.6)		Papillary (Neg)
A	Papillary (65)	E19del, T790M	Papillary (E19del, 22.7; T790M, 33.6)	40	Papillary (20% pos)
B	Acinar (30)		Acinar (E19del, neg; T790M, neg)		Acinar (Pos)
C	Microp (5)		Microp (E19del, 5.4; T790M, 1.7)		Microp (Neg)
A	Lepidic	L858R, T790M	Lepidic (L858R, 33.5; T790M, 30.5)	0	0

### Intratumoral heterogeneous *EGFR* status and expression of PD-L1 in LACs

Among the eighteen T790M-positive LACs, 10 LACs had acinar pattern and 6 of them were positive with PD-L1. For these 18 patients, each pattern had been captured and detected by ddPCR to confirm the intratumoral T790M abundance. Nine LACs consisting of more than one pattern had heterogeneous *EGFR* status and *EGFR* wild-typed components were positive for PD-L1 expression. We noticed that acinar component was apt to express PD-L1 in a tumor mass and micropapillary and papillary more frequently had both sensitizing and T790M mutation, meanwhile, less often overexpressed PD-L1 protein. We found that PD-L1 expression occurred in acinar and solid areas, but not in micropapillary area (Fig. 5). Cancer cells from different areas in a tumor mass possessed different *EGFR* statuses and PD-L1 expression. As shown in Fig. 5c-e, both areas I and II from one tumor were negative both for E19del and T790M. However, area III from the same tumor coaltered with both E19del and T790M with different abundance. Similar results were observed in other tumors (Table 3). Eight of 10 *EGFR*-mutated LACs lacking PD-L1 protein were found with single components. However, *EGFR* mutated components in composite LACs, although in part, still expressed PD-L1 (Table 3). Discordant *EGFR* status and PD-L1 expression suggested that a tumor mass harbored genetic aberration.

## Discussion

Lung adenocarcinomas frequently occur in ‘mixed pattern’ and percentages (up to 5%) of various histological components: acinar, papillary, micropapillary, lepidic and solid, are evaluated by semiquantitative assessment and should be reported according to the new WHO classification [1]. It is crucial to adopt a practical way to address tumors comprised of a complex histological constitution, since 70 to 90% of surgically resected lung cases were diagnosed invasive adenocarcinomas. Prominent diverse patterns in morphology and heterogeneity in biology among adenocarcinomas are paid more and more attention by pathologists upon the establishment of the new classification. We commenced our study after we reviewed all the sections and renewed diagnoses based on the new classification. Articles on the topic of micropapillary AC have reported patients in early-stage with a poor prognosis [10, 11]. It has recently been convinced that tumors classified as micropapillary also have a poor prognosis similar to adenocarcinomas with a predominant solid subtype [12].

This study discussed the association between LAC histological subtypes, PD-L1 expression levels and primary resistance to *EGFR*-TKIs. Our research demonstrated that PFS time of LAC patients exhibited a better prognosis among patients with TPS < 1%, than those with TPS > =50% (22.9 months versus 15.3 months). Since that LAC usually harbor more than one components in its parenchyma, we compared the PFS of different PD-L1 expression level with histological constitution. Patients harboring two histological components and with TPS < 1% had longer PFS time than those with TPS > =50% (25.5 months versus 13.6 months). However, prognosic significance was not evinced among LACs with single or > =three components. PD-L1 was not homogeneously expressed even in a tumor mass (Fig. 1b and Table 3). We also observed that heterogeneous PD-L1 expression, especially in the group of TPS 1–49%, was inclined to the tumor cells in the rim of tumor population against stroma (Fig. 1d). Given the heterogeneity of PD-L1 expression, it is important to understand the signals that induce the expression of PD-L1 on tumour cells. Two general mechanisms for the regulation of PD-L1 by tumour cells have emerged: innate immune resistance and adaptive immune resistance [13]. For this reason, we observed PD-L1 expression in different LAC subtypes and compared the effect on patients’ prognosis. Acinar LACs with TPS < 1% had longer PFS time compared with those with higher PD-L1 level. Likewise, solid LACs had a similar prognosis between the groups of TPS < 1% and TPS 1–49% even if it was not significant. K. Yoshimura, et al [14] reported that PD-L1 are also heterogeneously expressed in the same primary tumor tissue in a patchy pattern, as shown in our study (Fig. 1c and Fig. 5b). Histological subtypes were really associated with heterogeneous PD-L1 expression and patients’ prognosis.

ITH is attractive depending on recent technological advances in higher resolution and more rapid analysis on cancer genomes. Intratumoral heterogeneity is considered either the molecular features or the pathologic features of LAC. However, few studies have focused on the relationship between the two. In order to better address the current controversies and to conceive future directions, it seems apposite to investigate thoroughly the early motivations for targeting the PD-1/PD-L1 axis in *EGFR*-mutated NSCLC. This concept was in part recommended by retrospective studies prompting frequent PD-L1 expression in *EGFR*-mutated NSCLC [15, 16]. The co-occurrence of PD-L1-positivity and activating *EGFR* mutations in clinical NSCLC specimens was first reported based on 164 surgically resected samples [16]. Evidence to support this notion is that PD-L1 is a downstream target of *EGFR* signaling, and this is interceded through IL-6/JAK/STAT3, NFĸB and p-ERK1/2/p-c-Jun pathways [17–19]. PD-L1 expression can be repressed by *EGFR*-TKI [18]. In contrast, one study found PD-L1 expression was increased following gefitinib treatment [20]. However, the conclusions from previous studies are drawn based on the analysis on tumor lesion rather than on each histological component. What potential impact of *EGFR* mutated cancer cells expressing PD-L1 in tumor cell population remains be illuminated. A striking finding of our study is the identification of intratumoral genetic heterogeneity in LAC that harbors *EGFR* heterogeneous alterations and differential PD-L1 expression. In spite of *EGFR* status, PD-L1 overexpression occurred in LACs with solid (47.6%) versus those without this pattern, whereas low level expression of PD-L1 in mucinous ACs. Zito Marino, F. et al reported that PD-L1 expression was more frequently in LACs with solid pattern [21]. In addition, PD-L1 expression was not homogeneous in a tumor parenchyma and more common in heterogeneous pattern. Therefore, we further investigated and analyzed the relevance of *EGFR* statuses and PD-L1 expression in the 18 LACs with sensitizing/T790M mutation. We noticed that PD-L1 expressed more frequently in those components without *EGFR* mutation, especially in acinar areas, but really overexpressed in *EGFR*-mutated components. We suspect that *EGFR*-mutation driven PD-L1 expression are activated through the pathways as reported before [22]. In our study, LACs with single components harboring *EGFR* mutation tended to be short of PD-L1 expression. It seemed to be paradoxical with previous study that the biological association is indirectly consolidated by coexistence of PD-L1 upregulation in *EGFR*-mutant NSCLC, as observed in retrospective studies [16, 19], but not supported in subsequent pooled analysis [22]. However, another cancer cell-centric mode of PD-L1 upregulation is considered as adaptive immune resistance [23], which is characterized by an increased expression of PD-L1 on tumor cells and immune cell subpopulation in reaction to robust CD8+ T-cell-mediated immunosurveillance. Adaptive PD-L1 upregulation relies on effective immunorecognition, which is promoted by an increased somatic mutational and neoantigen burden. However, for hitherto indefinite reasons, mutational burden seems to be lower in *EGFR*-driven tumors [24]. We consider that this dilemma results from the ITH of LACs. The fact that cancers with *EGFR* mutation partially expressed PD-L1 protein suggests that a tumor mass seems to exist as if intercoursed subpopulations of cancer cells present with different biological behavior, both in PD-L1 expression and *EGFR* mutation. These attributes enable LACs to represent a heterogeneous response to clinical therapy. It is also manifested that histological subtypes really prompt the possibility of potential resistance to both *EGFR*-TKI and PD-L1-related immunotherapy.

A striking finding of this study is the identification of intratumoral genetic heterogeneity in LACs harboring driver coalterations. 17 patients was identified with co-occurrence of *EGFR* sensitizing and resistant mutations providing an incidence rate of 6.5%. Because it is unclear whether sensitizing and resistant mutations coexist in same or different tumor cells, we used LCM to capture pure tumor cells within both the same and different growth patterns. With this method, we found that *EGFR* mutation types did not concomitantly coexist in all tumor cells. Interestingly, *EGFR* mutations by ddPCR assay showed that abundance of these mutations were different in the same cell population. Therefore, we determined a difference in the driver status among spatially separated tumor areas. In particular, the relative abundances of the two altered genes were different in the same tumor areas, which suggests that oncogenic genetic profile may not be the same in all tumor cells within a same primary tumor (Fig. 5c-e). To fully recognize morphologic heterogeneity, we observed that the abundances of sensitizing and T790M mutation were not concordant in micropapillary and papillary components. We also observed similar results by using ARMS according to cycle threshold value. Intratumoral heterogeneity of *EGFR* mutations is demonstrated associated with the distribution of histological components in mixed LACs [25]. We also revealed that intratumoral heterogeneity of *EGFR* mutations really existed in the same histological subtypes of LAC. Therefore, we speculate that clone evolution, not only histological heterogeneity, may be mainly responsible for molecular intratumoral heterogeneity of LAC. The findings of intratumoral heterogeneity in *EGFR* sensitizing/T790M coaltered LAC may be hypothesized by Darwinian-like clonal evolutionary dynamics and the resulting complex clonal architecture of LAC as reported by Cai et al [26]. In this study, tumor cells with dual altered driver genes occurring mainly in micropapillary and papillary components prompt that these components most likely harbor heterogeneous clone. Previous studies have shown that a substantial proportion of malignant tumors have a multiclonal signature [27]. The fact that T790M clones were selected by the TKI therapy support this hypothesis, because T790M occurs in 60% patients upon *EGFR*-TKI therapy [28, 29]. Differential expression of PD-L1 in a tumor mass seems to testify this speculation as well. Discordant PD-L1 expression in a tumor mass is thereof suspected to reactive/adaptive expression owing that it usually occurs among the peripheral cancer cells against stroma. Resistance to TKIs is considered one of the unknowns of cancer; therapy selection may make tumors become more heterogeneous, which may be the major reason for resistance to TKIs [30]. The complex dynamics of clonal evolution could produce unique and unpredictable patterns of clonal architecture that are spatially and temporally heterogeneous [31, 32]. Clonal evolution underlying tumor progression probably proceeds in a branching, rather than in a linear manner, which might result in substantial clonal diversity that accordingly contributes to genetic heterogeneity within tumors [32]. Importantly, our findings may provide a rationale more reasonable to treat patients with dual mutations with Osimertinib. In addition, any pathologic diagnosis based on a whole mass does not adequately determine the oncological heterogeneity. Analyses on the correlation of histological subtypes and *EGFR* mutation show that *EGFR* mutation were significantly more common in LACs with micropapillary, papillary and lepidic pattern and less common in those with mucinous pattern. However, considering the potential impact of genetic intratumoral heterogeneity on histological feature, especially in LAC with more than one histological component, the situation may be more intricate than it looks.

## Conclusion

Our study provided additional evidence for the notion that concomitant *EGFR* sensitizing/TKI-resistant mutation and PD-L1 expression could limit the clinical prognosis in LAC patients received anti-PD-L/*EGFR* TKI-based therapies. However, it should be emphasized that our data, together with previous studies, facilitate the potential to promote the combination of *EGFR*-TKI and immune checkpoint inhibitor in some specific LAC subtypes, such as those with solid pattern. The findings from this study that patients of T790M coaltered with *EGFR* sensitizing mutation should have a better response to the third-generation *EGFR*-TKI (Osimertinib) combined with immune checkpoint inhibitor. However, the algorithm of therapeutics to LACs should be defined relying on more studies on the components of LACs. Intratumoral heterogeneity of oncogenic drivers in LACs should be taken seriously, because they can hamper precise molecular diagnosis and selection of the most appropriate treatment in clinical practice. These findings should be viewed with caution in clinical practice, suggesting that the use of gefitinib should be limited to patients with micropapillary or papillary, but Osimertinib will be a better candidate for *EGFR*-mutated LACs. The explorations in clinical setting are urgent and promising.

## Data Availability

All data present and analyzed during this study are included in this published article.

## Abbreviations

*EGFR*-TKI: Epidermal growth factor receptor tyrosine kinase inhibitors
ITH: Intratumoral heterogeneity
LAC: Lung adenocarcinoma
LCM: Laser capture microdissection
MR: Mixed responses
NSCLC: Non-small cell lung cancer
